# Investigating Microtubule-Associated Protein 2 in the Study of Postoperative Delirium

**DOI:** 10.56392/001c.155608

**Published:** 2026-01-27

**Authors:** Tina McKay, Occam Kelly Graves, Anna Toombs, Christopher Simon, Oluwaseun Akeju

**Affiliations:** 1 Anesthesiology, Massachusetts General Hospital,; 2 Anesthesiology, Brigham and Women’s Hospital

**Keywords:** neurodamage, delirium, traumatic brain injury, single-molecule immunoassay, risk biomarker, dementia

## Abstract

**Background:**

This study explored potential associations between microtubule-associated protein 2 (MAP2), as a marker of neuronal stress, and postoperative delirium.

**Methods:**

Custom single-molecule immunoassays were used to measure MAP2 in serum collected from two small cohorts of older adults undergoing major cardiac surgery. An in vitro study of differentiated SH-SY5Y cells derived from a neuroblastoma cell line was performed to assess MAP2 secretion following exposure to acute glutamate stimulation.

**Results:**

Patients who experienced postoperative delirium within 3 days of surgery had increased MAP2 serum levels on postoperative day 1. Perioperative blood sampling revealed an increase in circulating MAP2 at the end of cardiopulmonary bypass that was sustained up to postoperative day 3 in patients who developed delirium. Differentiated SH-SY5Y cells exposed to high-dose glutamate showed increased MAP2 in conditioned media independent of changes in total cytosolic protein levels suggesting that extracellular stimuli may promote acute secretion of MAP2 without inducing cell rupture.

**Conclusion:**

Further studies exploring the MAP family as biomarkers of cognitive changes following surgery are warranted.

## INTRODUCTION

Microtubule-associated proteins (MAPs) provide critical support to the neuronal cytoskeleton and mediate intracellular transport and migration. Growing evidence suggests that MAPs may be involved in the dynamics underlying dendritic and axonal retraction during neurodegeneration.^1^ The most infamous MAP isoform, tau, is an established biomarker of dementia and cognitive dysfunction^2,3^ and has been previously associated with postoperative delirium.^4–7^ Of the four major families of MAPs, MAP2 has been understudied as a biomarker of neurological conditions but is highly enriched in the brain compared to the periphery^8^ and serves a critical role in preserving neuronal structure, neurogenesis, and neuroplasticity.^9,10^ We hypothesize that blood MAP2 levels may reflect dynamic changes occurring in the brain in response to major cardiac surgery and indicate risk of cognitive dysfunction. Here, we developed custom single-molecule immunoassays to detect MAP2 in serum and investigate potential associations with the development of postoperative delirium and neuronal stress.

## METHODS

### CLINICAL STUDY

All studies were approved by the Mass General Brigham Institutional Review Board (Protocol #: 2018P000480 and 2022P000445). Serum samples derived from subjects undergoing major cardiac surgery were analyzed from 2 independent cohorts.^11,12^ Cohort 1 was composed of 19 subjects with serum collected before surgery and on postoperative day 1. Cohort 2 was composed of 57 subjects with serum collected at 6 perioperative timepoints: immediately before surgery, at the start and end of cardiopulmonary bypass, and on postoperative days 1 – 3. Inclusion criteria for both cohorts included older subjects (⩾ 60 years) scheduled for major cardiac surgery with cardiopulmonary bypass and postoperative cardiac ICU admission to permit blood collection. Exclusion criteria included blindness, deafness, and the inability to speak English, renal or liver failure, COVID-symptomatic or positive, > 2 days in the ICU in the month prior to surgery, and severe neurocognitive damage. Delirium was assessed twice daily using the Confusion Assessment Method by trained clinical research staff.^13^ We have previously published serum analysis of Cohort 1^6^ and Cohort 2.^12,14^

### BIOMARKER ANALYSIS

The Simoa homebrew technology^15^ was utilized to generate custom Simoa plates using validated paired antibodies for binding and detection of MAP2 (Supplemental Methods).

### SH-SY5Y STUDY

Differentiated SH-SY5Y cells derived from a neuroblastoma cell line were cultured *in vitro* and stimulated with acute glutamate to induce excitotoxicity (Supplemental Methods).^16^

### STATISTICAL ANALYSES

Effect estimates are presented as fold-change or median difference and their associated standard deviation or 95% confidence intervals (CIs). No imputation of missing data was performed, and only datapoints outside of the limits of quantification were excluded. No adjustments for covariates were made due to the small sample sizes and risk of overfitting. To identify possible associations with delirium, the values were log-transformed and normalized to the preoperative MAP2 concentration comparing no delirium and delirium groups using a Mann-Whitney U test. The normalized fold-change in MAP2 concentrations at each timepoint from baseline was assessed with a Wilcoxon matched-pairs signed rank test. Statistical analysis for the MAP2 *in vitro* studies was based on a Brown-Forsythe and Welch’s ANOVA test. For all analyses, two-sided p-values < 0.05 were considered statistically significant.

## RESULTS

**Figure 1.**
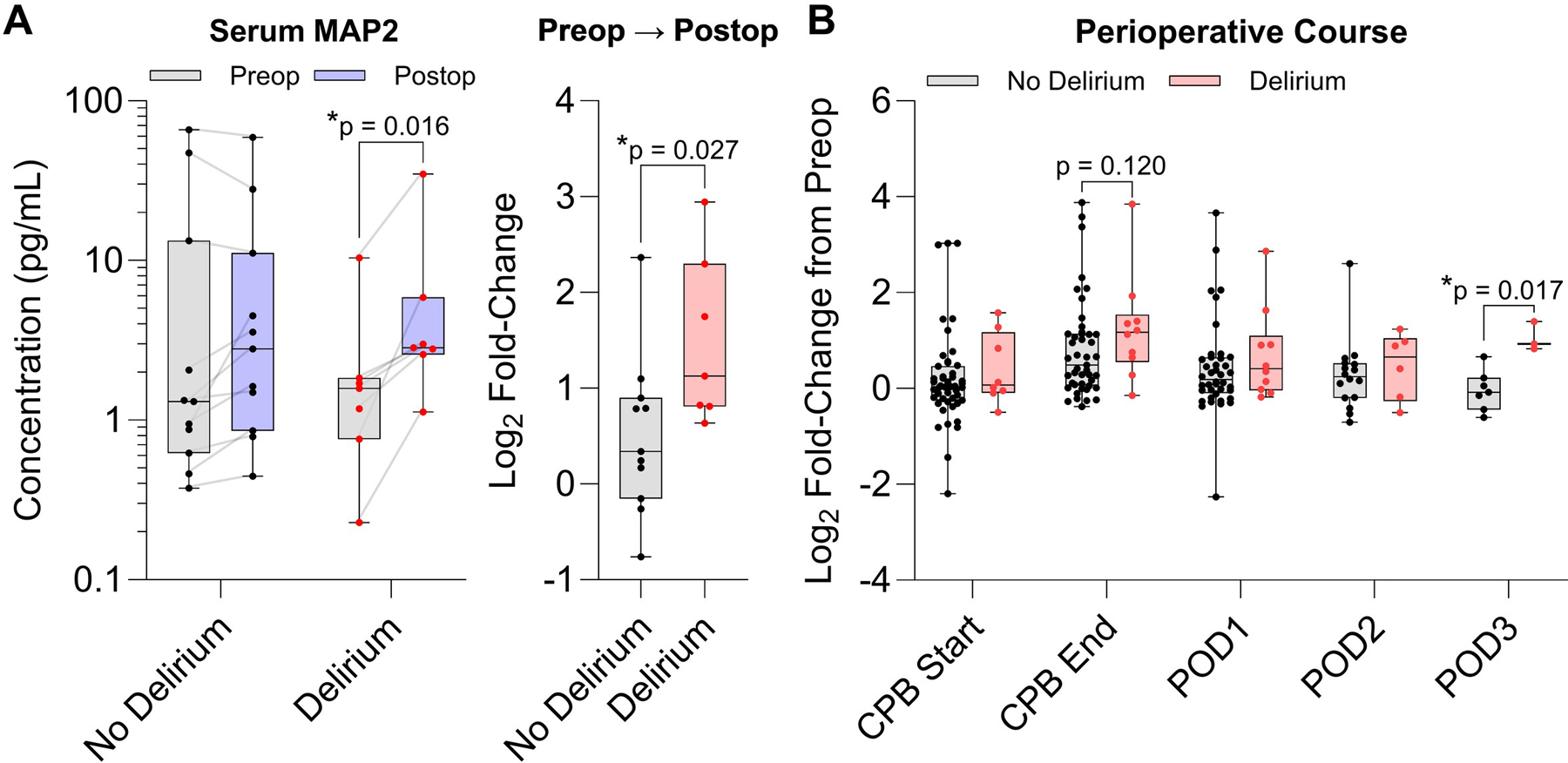
MAP2 concentrations in serum collected from 2 independent cohorts of adults aged 60 years and older undergoing major cardiac surgery. (**A**) Preoperative and postoperative day 1 MAP2 levels in Cohort 1 stratified by timepoint and delirium status (*left*), and the fold-change in MAP2 from preop to postoperative day 1 (*right*). Statistical significance evaluated using a Wilcoxon-matched signed rank test (*left*) and a Mann-Whitney U test (*right*) (n = 11 no delirium and n = 8 delirium; 1 patient with delirium had values above the assay range). (**B**) Perioperative timecourse of MAP2 levels in Cohort 2 normalized to preoperative values (n = 47 no delirium and n = 10 delirium). Statistical analysis based on multiple Mann-Whitney U tests. (*Abbreviations*: CPB, cardiopulmonary bypass; POD, postoperative day).

Sera collected from two independent cohorts composed of patients aged 60 years and older undergoing major cardiac surgery with cardiopulmonary bypass at Massachusetts General Hospital were analyzed (Table 1). In general, circulating MAP2 levels increased after surgery by postoperative day 1 compared to baseline (Supplemental Figure 1 – 2). In Cohort 1, patients who experienced postoperative delirium showed significantly higher MAP2 from preoperative values (2.74-fold, 95% CI [1.66 – 5.09], p = 0.016, n = 8) and a larger change to postoperative day 1 (1.94-fold, 95% CI [1.02 – 3.89], p = 0.027) compared to patients who did not develop delirium (n = 11, Figure 1A and Supplemental Table 1). Correlation analysis revealed a positive association between preoperative levels of MAP2 and P-Tau-231, which has been reported as a predictive biomarker of Alzheimer’s disease^17^ (Supplemental Figure 3). In Cohort 2 with intraoperative blood sampling, MAP2 levels peaked at the end of cardiopulmonary bypass and gradually lowered up to postoperative day 3 in patients who did not develop delirium (Supplemental Figure 4). Subjects who experienced delirium showed a modestly higher increase in MAP2 at the end of cardiopulmonary bypass (1.55-fold, 95% CI [−0.40 – 3.45], p = 0.12, n = 8) and postoperative day 3 (3.59-fold, 95% CI [0.91 – 6.09], p = 0.017, n = 3) relative to preoperative levels compared to patients who did not develop delirium (n = 45 and n = 7, Figure 1B and Supplemental Table 2). In both cohorts, delirium incidence was more strongly associated with the postoperative change in MAP2 from baseline values, rather than preoperative or postoperative timepoints alone, suggesting that the increase in serum MAP2 may reflect a vulnerability to surgical stressors.

**Figure 2.**
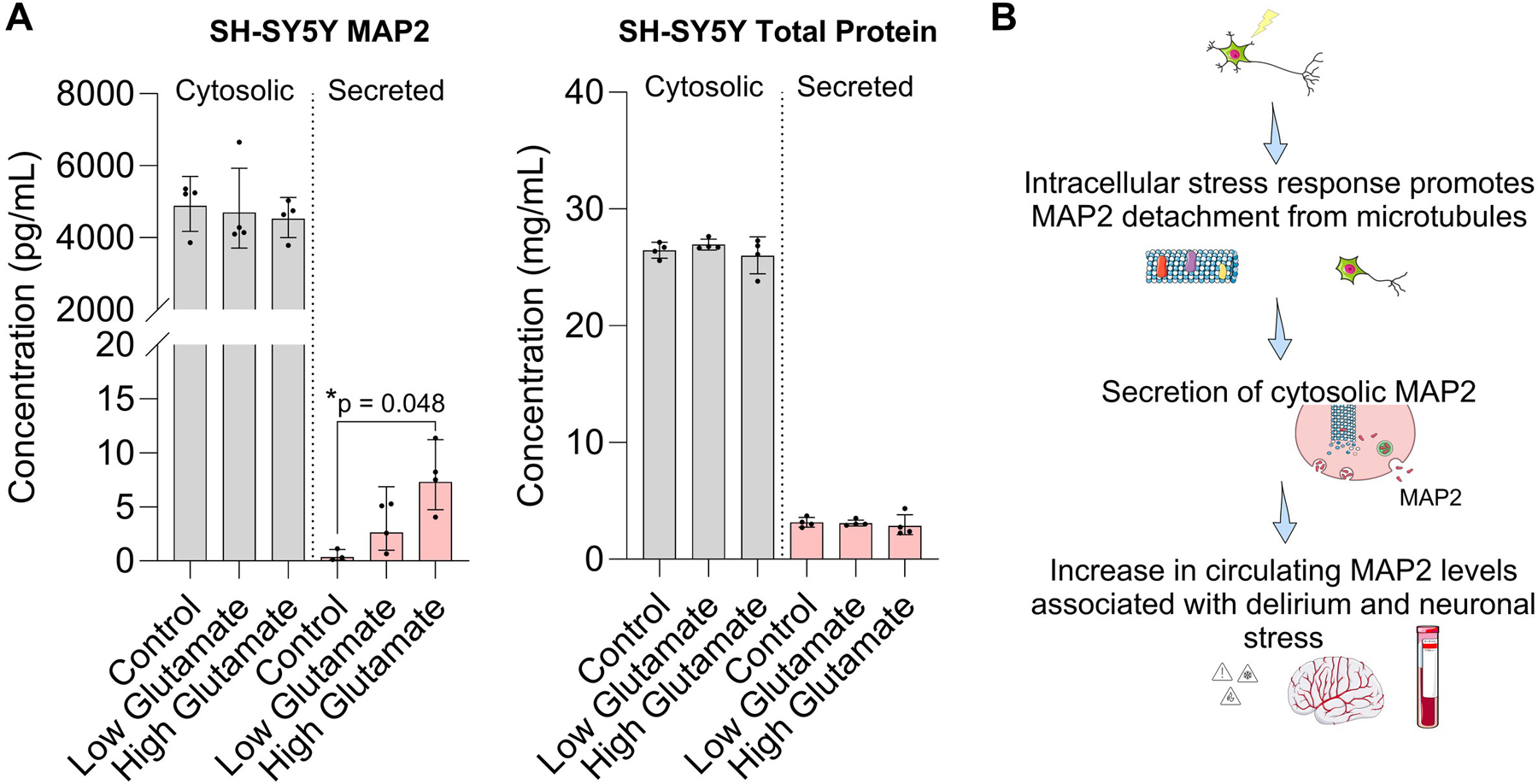
Mechanistic *in vitro* study using SH-SY5Y cells to study MAP2 secretion following glutamate stimulation. (**A**) MAP2 levels in differentiated SH-SY5Y cells stimulated with low-dose (50 mM) and high-dose (100 mM) glutamate. Cell lysates and media were isolated at 3 hours post-treatment and analyzed for MAP2 (*left*) and total protein levels (*right*). Statistical analysis based on Brown-Forsythe and Welch’s ANOVA tests. Data shown as mean ± standard deviation (n = 4 per group). (**B**) Schematic depicting the proposed mechanism involving MAP2 release following neuronal stress.

To provide mechanistic insight into extracellular MAP2 as an indicator of neuronal stress, we examined MAP2 secretion using an *in vitro* model of differentiated SH-SY5Y cells stimulated with glutamate to induce excitotoxicity. Extracellular MAP2 levels in the media significantly increased in a dose-dependent manner following acute glutamate exposure (13.55-fold increase, 95% CI [1.47 – 125.20], p = 0.048) with no significant change in total cytosolic protein levels, suggesting that MAP2 may be selectively secreted in the absence of overt cell lysis (Figure 2A). Our working hypothesis is that surgical stress promotes release of MAP2 from microtubules, leading to dendritic retraction and secretion of unbound, cytosolic MAP2 into the extracellular space and increased circulating levels in blood (Figure 2B). Collectively, our results lend support to further studies evaluating the use of circulating MAP2 as a surrogate marker of acute neurotoxicity and postoperative delirium.

## CONCLUSION

Delirium remains a relatively common complication of major cardiac surgery. The discovery and validation of sensitive blood-based biomarkers associated with postoperative delirium may enable the development of targeted interventions to promote cognitive resilience. This study developed and optimized custom assays to quantify the abundance of MAP2 in two independent cohorts of older adults undergoing cardiac surgery and identified possible associations with delirium based on quantitative protein measures. We selected MAP2 as a starting biomarker with unexplored potential given its specificity for the brain and hypothesized that its localization primarily to neuronal dendrites may provide a targeted picture of cognitive changes in response to surgical stress. The multifactorial pathophysiology involved in postoperative delirium has made it difficult to identify a therapeutic approach to improve resilience to surgical stressors. The ability to identify patient susceptibility to delirium using objective biomarkers, such as MAP2, may provide a means to assess whether targeted interventions are effective. Our results show that MAP2 protein levels increase in serum after major cardiac surgery and may be associated with postoperative delirium. The findings support our previous studies evaluating tau and neurofilament light chain as markers of neurodamage and possible associations with delirium.^6,12^ Longitudinal studies evaluating circulating MAP2 as a predictive marker of long-term cognitive changes may aid in defining its clinical utility in the context of established biomarkers of Alzheimer’s disease and related dementias. Understanding how changes in the blood-brain barrier may influence serum MAP2 levels and their association with cognitive outcomes may also be important areas to explore. The small samples sizes in our study are a notable limitation. Further studies validating these findings in larger, more diverse cohorts are required to adjust for confounders including age, sex, and baseline cognition. This short research report provides evidence that MAP2 may be a novel biomarker of postoperative delirium and is released from neuronal-like cells upon exposure to glutamate highlighting the need to explore the MAP family beyond tau protein in the study of delirium.

## Supplementary Material

Supplemental Materials

Download: https://deliriumjournal.com/article/155608-investigating-microtubule-associated-protein-2-in-the-study-of-postoperative-delirium/attachment/326670.pdf

## Figures and Tables

**Table 1.** Characteristics of 2 independent study cohorts composed of older adults aged 60 years and older undergoing major cardiac surgery. Continuous variables are shown as median [interquartile range], and categorical variables are shown as number (percentage).

Characteristic	Cohort 1		Cohort 2	
	No Delirium (n = 11)	Delirium (n = 8)	No Delirium (n = 47)	Delirium (n = 10)
Age (years)	70 [67 – 77]	73 [65 – 75]	69 [65 – 74]	73 [69 – 77]
Male Sex	8 (73%)	5 (63%)	40 (85%)	7 (70%)
Baseline T-MoCA Score (points)	18 [15 – 20]	19 [14 – 20]	19 [17 – 20]^†^	19 [15 – 21]
CPB Duration (minutes)	146 [92 – 216]	116 [97 – 161]	172 [126 – 191]	179 [139 – 277]

† 2 subjects with missing T-MoCA scores at baseline. (*Abbreviations*: CPB, cardiopulmonary bypass; T-MoCA, telephonic-Montreal Cognitive Assessment).
