# Supplementary material for: Investigating Microtubule-Associated Protein 2 in the Study of Postoperative Delirium
Source: Delirium (Bielef). Author manuscript; Available in PMC 2026 Feb 21. (PMC12922626; doi:10.56392/001c.155608)
Supplement: Supplemental Materials [file NIHMS2146701-supplement-Supplemental_Materials.pdf]

SUPPLEMENTAL MATERIALS

Supplemental Tables.

Supplemental Table 1. Statistical analysis for MAP2 associations and delirium in Cohort 1 comparing preoperative and postoperative day 1 values.

Supplemental Table 2. Statistical analysis for MAP2 associations and delirium in Cohort 2 comparing perioperative values at 6 timepoints in delirium and no delirium groups.

Supplemental Figures.

Supplemental Figure 1. Preoperative and postoperative day 1 serum levels in Cohort 1.

Supplemental Figure 2. Serum MAP2 levels in Cohort 2.

Supplemental Figure 3. Spearman correlation of preoperative serum levels of MAP2 and P-Tau-231 in Cohort 1 (red dots denote subjects who developed delirium).

Supplemental Figure 4. Perioperative MAP2 levels in subjects with blood collected at preop, CPB start and end, and postoperative days 1 – 3 in Cohort 2.

Supplemental Figure 5. RT-PCR analysis of differentiated SH-SY5Y cells treated with low-dose (50 mM) and high-dose (100 mM) glutamate for 3 hours *in vitro*.

Supplemental Figure 6. MAP2 calibration curve generated using a recombinant MAP2 protein standard of known concentration.

Supplemental Figure 7. Interplate variability comparing serum samples analyzed sequentially on separate plates.

Supplemental Figure 8. MAP2 concentration of standard human serum compared to SH-SY5Y cell lysate.

Supplemental Methods:

Biomarker Analysis
SH-SY5Y Study
Statistical Analysis

Supplemental References.

Supplemental Table 1. Statistical analysis for MAP2 associations and delirium in Cohort 1 comparing preoperative and postoperative day 1 values. A paired Wilcoxon signed-rank test was used to compare for postop and preop values and a Mann-Whitney U test for delirium and no delirium comparisons.

Description	Median Fold-Change	95% CI	P-Value
No Delirium (Postop vs. Preop)	1.389	0.995 to 2.076	0.0537
Delirium (Postop vs. Preop)	2.737	1.658 to 5.086	0.0156
Baseline Normalized (Delirium vs. No Delirium)	1.944	1.021 to 3.887	0.0268

(*Abbreviation*: CI, confidence interval).

Supplemental Table 2. Statistical analysis for MAP2 associations and delirium in Cohort 2 comparing perioperative values at 6 timepoints in delirium and no delirium groups. A paired Wilcoxon signed-rank test was used for comparisons between each timepoint to preop and a Mann-Whitney U test for delirium and no delirium comparisons.

Description	Median Fold-Change	95% CI	P-Value
No Delirium (CPB Start vs. Preop)	1.166	0.82 to 1.81	0.386
Delirium (CPB Start vs. Preop)	2.493	0.58 to 16.31	0.313
No Delirium (CPB End vs. Preop)	4.308	2.48 to 9.27	0.000000073
Delirium (CPB End vs. Preop)	12.692	3.42 to 114.58	0.00391
No Delirium (POD1 vs. Preop)	1.794	1.22 to 3.28	0.00305
Delirium (POD1 vs. Preop)	3.324	1.15 to 26.51	0.0371
No Delirium (POD2 vs. Preop)	1.611	0.82 to 3.18	0.193
Delirium (POD2 vs. Preop)	2.602	0.31 to 17.44	0.219
No Delirium (POD3 vs. Preop)	0.868	0.37 to 2.28	0.813
Delirium (POD3 vs. Preop)	10.565	6.78 to 25.06	0.25
**Description**	**Median Fold-Difference**	**95% CI**	**P-Value**
Baseline normalized CPB Start (Delirium vs. No Delirium)	0.510	-0.83 to 2.91	0.534
Baseline normalized CPB End (Delirium vs. No Delirium)	1.550	-0.4 to 3.45	0.12
Baseline normalized POD1 (Delirium vs. No Delirium)	0.673	-0.76 to 2.55	0.296
Baseline normalized POD2 (Delirium vs. No Delirium)	1.160	-1.9 to 2.91	0.329
Baseline normalized POD3 (Delirium vs. No Delirium)	3.594	0.91 to 6.09	0.0167

(*Abbreviations*: CI, confidence interval; CPB, cardiopulmonary bypass; POD, postoperative day).

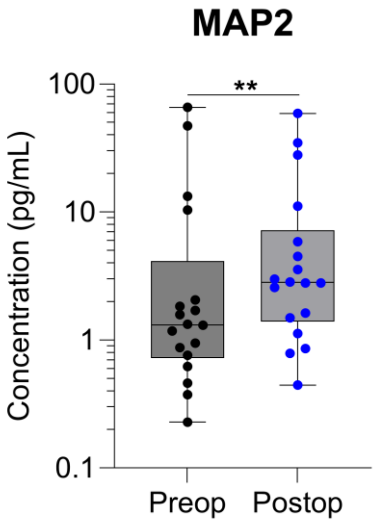

Supplemental Figure 1. Preoperative and postoperative day 1 serum levels in Cohort 1. Statistical analysis based on a ratio paired-T test assuming lognormality with ** = $p < 0.01$ (1 patient had preop and postop values above the assay range).

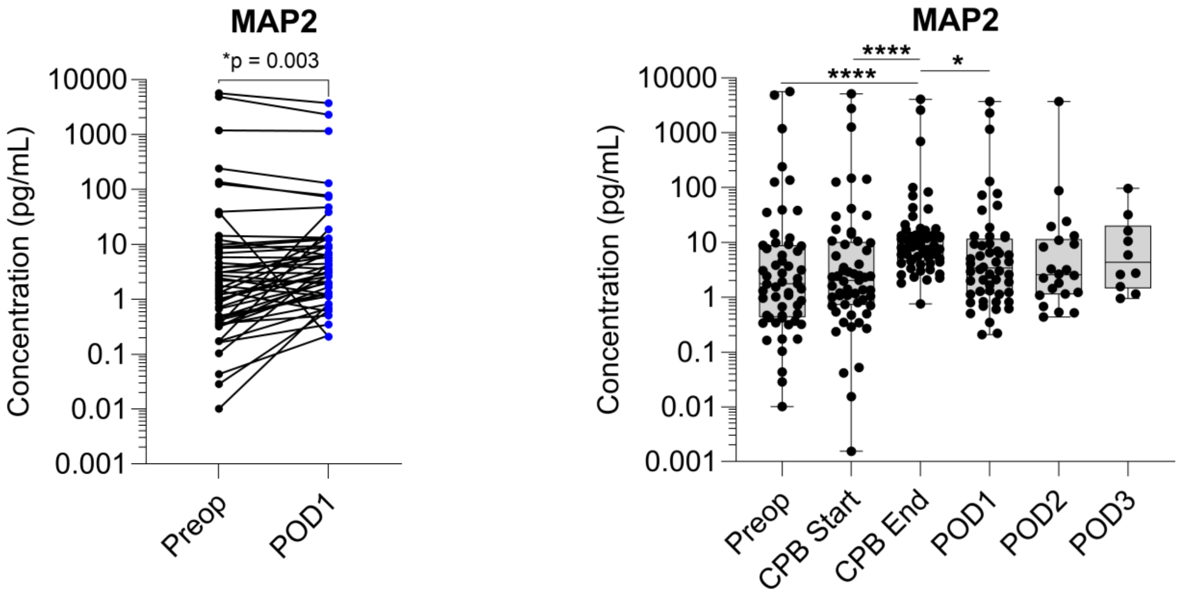

Supplemental Figure 2. Serum MAP2 levels in Cohort 2. Statistical analysis for the preop and POD1 comparison based on the ratio paired T test assuming lognormality. Statistical analysis for the 6 perioperative timepoints was based on the Kruskal-Wallis test with Dunn's multiple comparisons test with * = $p < 0.05$ and **** = $p < 0.0001$. (*Abbreviations*: CPB, cardiopulmonary bypass; POD, postoperative day).

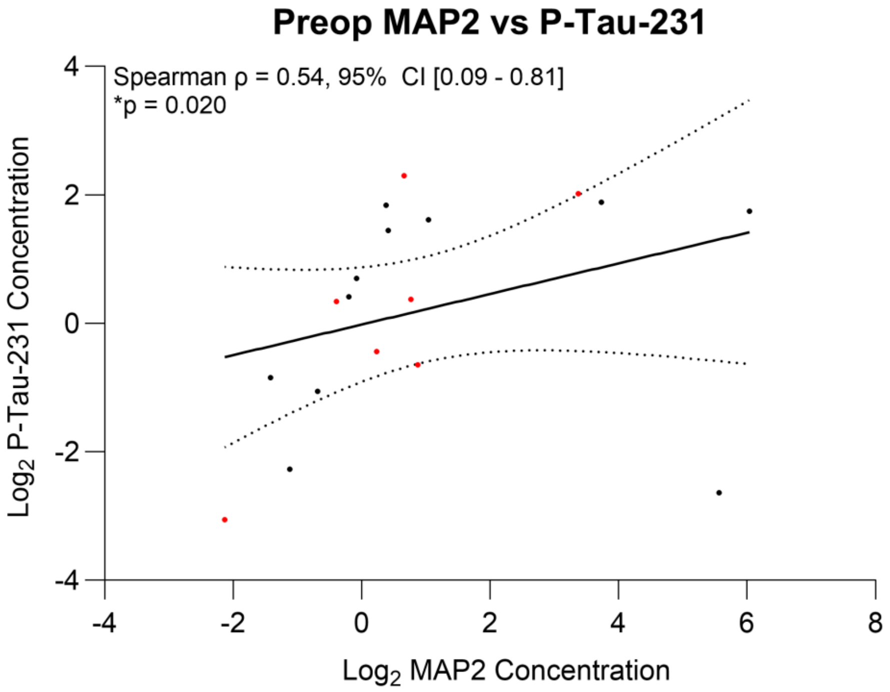

Supplemental Figure 3. Spearman correlation of preoperative serum levels of MAP2 and P-Tau-231 in Cohort 1 (red dots denote subjects who developed delirium).

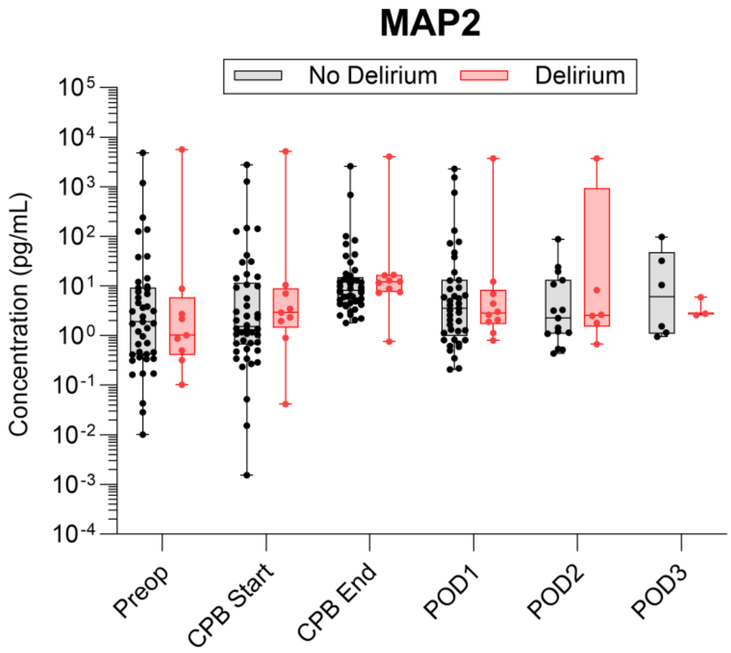

Supplemental Figure 4. Perioperative MAP2 levels in subjects with blood collected at preop, CPB start and end, and postoperative days 1 – 3 in Cohort 2. (*Abbreviations*: CPB, cardiopulmonary bypass; POD, postoperative day).

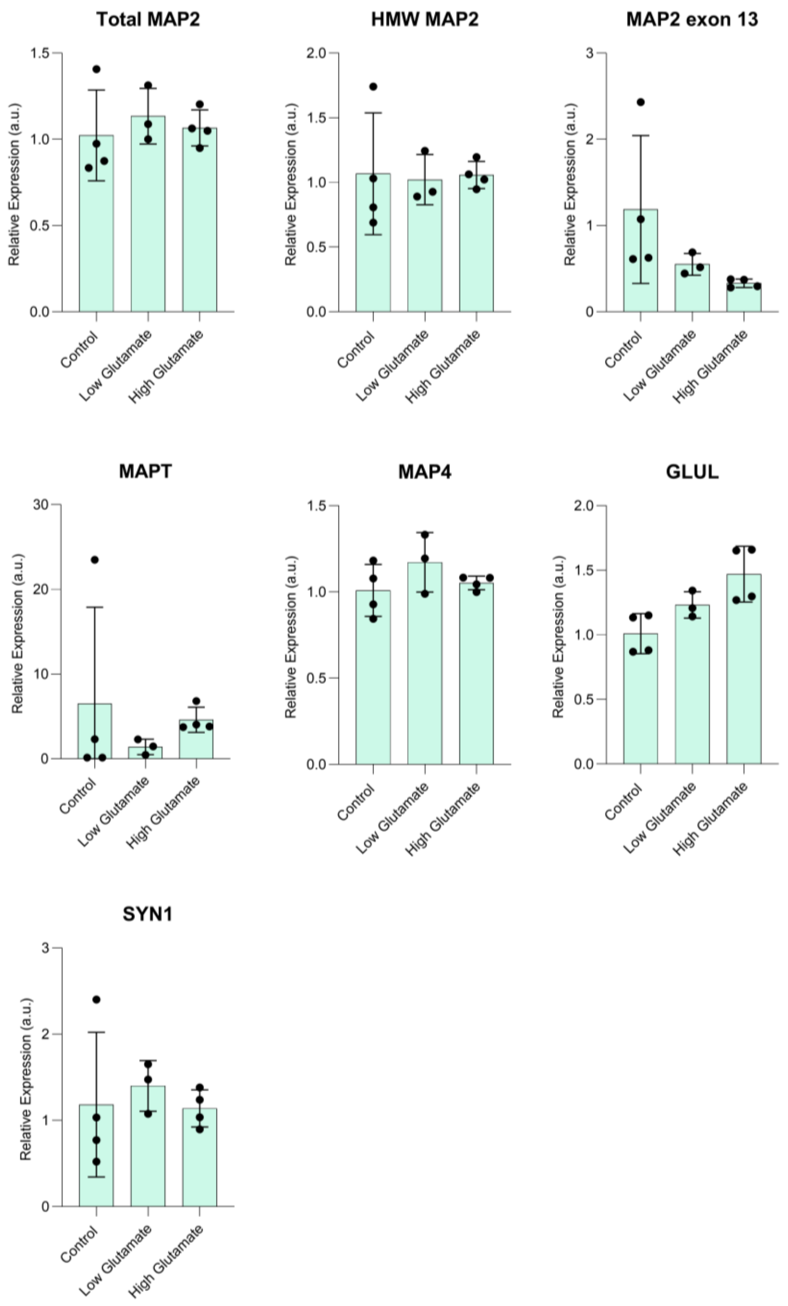

Supplemental Figure 5. RT-PCR analysis of differentiated SH-SY5Y cells treated with low-dose (50 mM) and high-dose (100 mM) glutamate for 3 hours *in vitro*. Gene expression was normalized to the housekeeping gene, GAPDH, and presented as relative expression determined using the ΔΔCt method. Data represented as mean ± standard deviation. (*Abbreviations*: HMW, high-molecular weight).

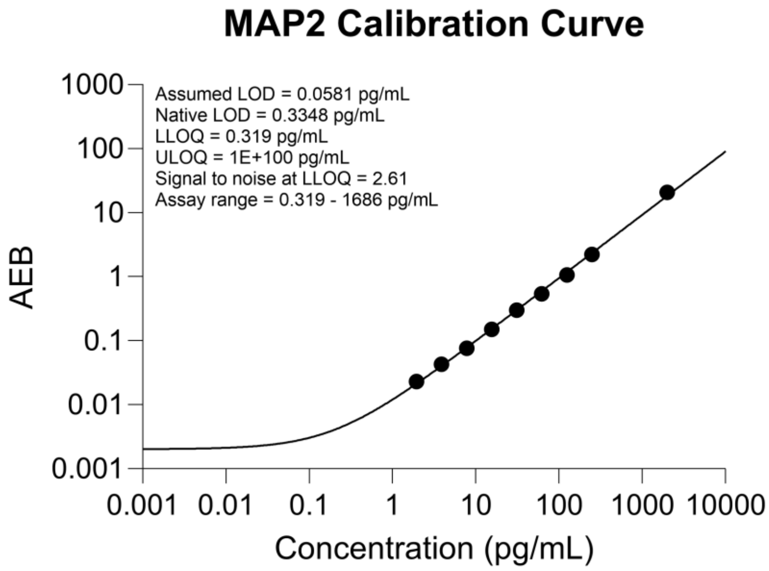

Supplemental Figure 6. MAP2 calibration curve generated using a recombinant MAP2 protein standard of known concentration. (*Abbreviations*: AEB, average enzymes per bead; LLOQ, lower limit of quantification; LOD, limit of detection; ULOQ, upper limit of quantification).

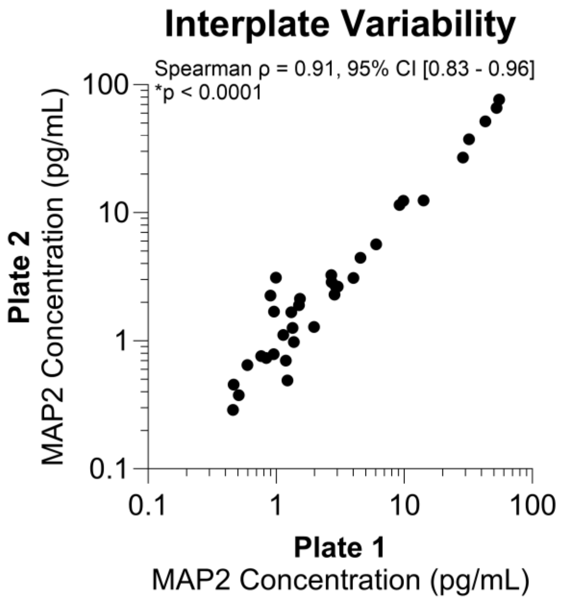

Supplemental Figure 7. Interplate variability comparing serum samples analyzed sequentially on separate plates (n = 35).

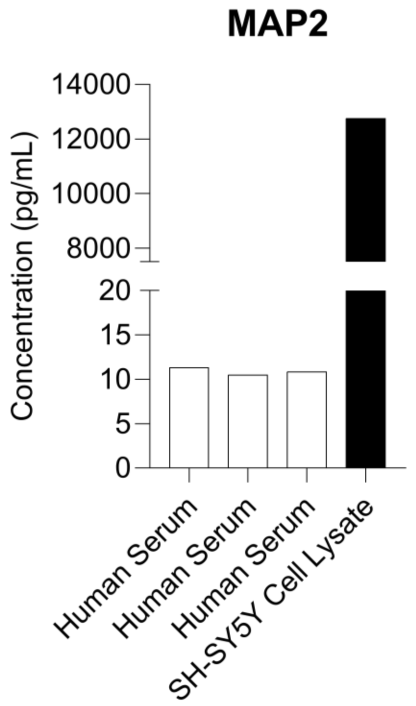

Supplemental Figure 8. MAP2 concentration of human serum compared to SH-SY5Y cell lysate.

Supplemental Methods:

Biomarker Analysis: The protocol for the Simoa Homebrew Assay was followed according to the manufacturer's protocol (Quanterix, Billerica, MA, USA). The following antibodies and standard protein were used: MAP2 Capture Antibody (monoclonal antibody, M13 clone; Vendor: ThermoFisher, Waltham, MA, USA; Item # 13-1500), MAP2 Detector Antibody (MT-08 clone; Vendor: ThermoFisher; Item # MA1-19426), MAP2 Standard (Human Microtubule Associated Protein MAP2 Projection P3; Vendor: EnCor Biotechnology, Gainesville, FL, USA; Catalog # Prot-r-MAP2AB-P3 × 1). A 3-step digital immunoassay protocol modified from the Simoa P-Tau-231 Advantage Kit Instructions was used to quantify the concentration of MAP2 in serum. CV Profiling was used to estimate the limit of detection, lower and upper limits of quantification, and the signal to noise at the lower limit of quantification from calibration curve data. P-Tau-231 was measured in serum following the manufacturer's protocol (Quanterix). All biomarker analyses were performed blinded to delirium status and timepoint to minimize bias. Samples below or above the level of quantification were excluded from the analysis.

SH-SY5Y Study: *Cell Culture* – SH-SY5Y cells were obtained from ATCC and cultured in Dulbecco's Modified Eagle Medium (DMEM, high glucose, no glutamine, Gibco, Waltham, MA, USA) containing 10% fetal bovine serum (FBS, Avantor, Waltham, MA, USA) and 100 units/mL of penicillin and 100 µg/mL streptomycin (1% of Penicillin-Streptomycin (10,000 U/mL), Gibco). The cells were seeded in a 6- or 12-well plate and maintained at 37°C and 5% CO_2 for 24 – 72 hours. Differentiation to a neuronal phenotype was performed using complete DMEM supplemented with 10 µM retinoic acid (CAS # 302-79-4, VWR, Radnor, PA, USA).[1] The cultures were then incubated for 5 – 7 days to permit differentiation, followed by stimulation with solutions of glutamate (L-glutamic acid monosodium salt hydrate, CAS # 142-47-2, Sigma Aldrich, St. Louis, MO, USA) prepared in complete DMEM followed by filter-sterilization (0.2 micron). Cultures were treated with glutamate or vehicle for 3 hours followed by media collection and cell isolation for protein or RNA extraction. M-PER lysis buffer (ThermoFisher) containing 1X protease inhibitor cocktail was used to extract protein from cell lysates. Protein concentrations were determined by Nanodrop (NanoDrop 2000, ThermoFisher) based on UV absorbance at 280 nm with 1 Abs = 1 mg/mL.

PCR Assay – Total RNA was extracted using the Quick-RNA Zymo Research Mini Kit following the manufacturer's protocol (Zymo Research, Tustin, CA, USA). A NanoDrop One device was used to determine RNA concentration and purity. Complementary DNA (cDNA) was synthesized from 500 ng of total RNA using the High-Capacity cDNA Reverse Transcription Kit (ThermoFisher). Quantitative Polymerase Chain Reaction (qPCR) was performed using 96-Well

Thermal Cycler with Applied Biosystems™ PowerUp™ SYBR™ green master mix (Applied Biosystems, Waltham, MA, USA). GAPDH was used to normalize targeted genes, and relative expression levels were calculated using the ΔΔCt method.[2] Each sample was run in duplicate. Results over one cycle difference were not included in the analysis.

Statistical Analysis: Aggregated preoperative and postoperative comparisons were assessed using a ratio paired-T test based on lognormality. Comparisons between perioperative timepoints were analyzed using the Kruskal-Wallis test with Dunn's multiple comparisons test.

Supplemental References.

1. Kovalevich J, Langford D. Considerations for the use of SH-SY5Y neuroblastoma cells in neurobiology. *Neuronal cell culture: methods and protocols*. Springer; 2013:9-21.
2. Schmittgen TD, Livak KJ. Analyzing real-time PCR data by the comparative CT method. *Nature protocols*. 2008;3(6):1101-1108.
